# Study on the Visible-Light Photocatalytic Performance and Degradation Mechanism of Diclofenac Sodium under the System of Hetero-Structural CuBi_2_O_4_/Ag_3_PO_4_ with H_2_O_2_

**DOI:** 10.3390/ma11040511

**Published:** 2018-03-28

**Authors:** Xiaojuan Chen, Ning Li, Song Xu, Hailong Wang, Yumin Cai

**Affiliations:** 1 School of Environment and Chemical Engineering, Foshan University, Foshan 528000, China; xjchen0218@163.com (X.C.); xuson@yeah.net (S.X.); nzhailongwang@163.com (H.W.); cym0171@126.com (Y.C.); 2 College of Marine Sciences, Sun Yat-sen University, Guangzhou 510275, China; 3 Key Laboratory of Soil Contamination Bioremediation of Zhejiang Province, Zhejiang A & F University, Hangzhou 311300, China

**Keywords:** CuBi_2_O_4_, Ag_3_PO_4_, H_2_O_2_, heterojunction, visible-light, stability

## Abstract

Two kinds of CuBi_2_O_4_/Ag_3_PO_4_ with different heterojunction structures were prepared based on the combination of hydrothermal and in-situ precipitation methods with surfactant additives (sodium citrate and sodium stearate), and their characteristics were systematically resolved by X-ray Diffraction (XRD), Brunauer–Emmett–Teller (BET), X-ray Photoelectron Spectroscopy (XPS), Scanning Electron Microscope (SEM)/ High-resolution Transmission Electron Microscopy (HRTEM), UV-vis Diffuse Reflectance Spectra (DRS) and Photoluminescence (PL). Meanwhile, the photocatalytic properties of the catalysts were determined for diclofenac sodium (DS) degradation and the photocatalytic mechanism was also explored. The results indicate that both of the two kinds of CuBi_2_O_4_/Ag_3_PO_4_ exhibit higher photocatalytic efficiency, mineralization rate, and stability than that of pure CuBi_2_O_4_ or Ag_3_PO_4_. Moreover, the catalytic activity of CuBi_2_O_4_/Ag_3_PO_4_ can be further enhanced by adding H_2_O_2_. The free radical capture experiments show that in the pure CuBi_2_O_4_/Ag_3_PO_4_ photocatalytic system, the OH^•^ and O_2_^•−^ are the main species participating in DS degradation; however, in the CuBi_2_O_4_/Ag_3_PO_4_ photocatalytic system with H_2_O_2_, all OH^•^, *h*^+^, and O_2_^•−^ take part in the DS degradation, and the contribution order is OH^•^ > *h*^+^ > O_2_^•−^. Accordingly, the photocatalytic mechanism of CuBi_2_O_4_/Ag_3_PO_4_ could be explained by the Z-Scheme theory, while the catalysis of CuBi_2_O_4_/Ag_3_PO_4_ with H_2_O_2_ follows the heterojunction energy band theory.

## 1. Introduction

Diclofenac sodium (DS) is a typical non-steroidal anti-inflammatory drug [1,2,3]. The usage of DS in China amounts to thousands of tonnes every year. Because of the strong water solubility of DS and its poor absorption by organisms, most of the DS intake by human and animals is excreted through feces and urine in the form of parent or active metabolites, finally flowing into the aquaculture wastewater and municipal wastewater [4]. Numerous studies have indicated that only less than 20% of DS can be removed from the traditional sewage treatment plant. Therefore, the DS, which enters the environment with effluent water, not only causes a serious poisoning effect on aquatic organisms, but also poses a significant threat to other living beings and human health through food chain accumulation [4,5]. Based on this, the effective degradation technologies for DS must be explored to control its continued contamination.

Photocatalytic technology had aroused widespread concern in the field of organic pollutant treatment due to the advantages of mild reaction conditions, fast reaction speed, high mineralization rate, and less secondary pollution [6,7,8,9,10]. Moreover, photocatalysts are the main body of photocatalytic technology, which play a decisive role in the photocatalytic activity of the reaction system [11,12]. To make full use of the visible light spectrum that occupies 43% of the clean solar energy, the current research and development mainly focuses on the visible light-responsive photocatalysts. Silver phosphate (Ag_3_PO_4_), which can absorb sunlight with wavelength less than 520 nm, is a visible light-responsive photocatalyst discovered in 2010 [11,13]. It exhibits high quantum yield and excellent photocatalytic activity. However, the Ag^+^ on the surface of Ag_3_PO_4_ can be easily reduced to be Ag^0^ (4Ag_3_PO_4_ + 6H_2_O + 12*h*^+^ + 12*e*^−^ → 12Ag^0^ + 4H_3_PO_4_ + 3O_2_) in the pure Ag_3_PO_4_ photocatalytic reaction system [11,14,15]. This is due to the slight solubility of Ag_3_PO_4_ in solution (solubility of 0.02 g/L) and the higher electrode potential of Ag/Ag^+^ than the conduction band potential of Ag_3_PO_4_, causing the photo-corrosion. The photo-corrosion destroys the stability of Ag_3_PO_4_ and decreases its photocatalytic activity, finally leading to the inactivation of Ag_3_PO_4_.

Related studies have shown that the hetero-structural photocatalysts, which can be constructed by combination of Ag_3_PO_4_ and other semiconductor materials, not only can promote the separation of photogenerated electron-hole pairs through belt matching with each other, but also can optimize the photocatalytic activity and stability of Ag_3_PO_4_ through transformation and being captured by electrons [16,17,18,19,20]. CuBi_2_O_4_ is another visible light-responsive photocatalyst, although its quantum yield is low, it poses good chemical stability, high conduction band position, and strong reducing ability [21,22,23]. Currently, CuBi_2_O_4_ is mainly used for fluorescent and photocathode materials [24,25], and its application in the field of photocatalytic oxidation technology has been rarely reported. Based on the band structure characteristics of Ag_3_PO_4_ and CuBi_2_O_4_, heterojunction photocatalyst of CuBi_2_O_4_/Ag_3_PO_4_ should have excellent photocatalytic properties compared with the pure catalysts, including activity and stability.

Therefore, in this study, a combination of hydrothermal and in-situ precipitation methods were used to construct two kinds of hetero-structural photocatalysts CuBi_2_O_4_/Ag_3_PO_4_ by adding different surfactants of sodium citrate or sodium stearate in the preparation systems, which can be respectively named as CuBi_2_O_4_/Ag_3_PO_4_-SC and CuBi_2_O_4_/Ag_3_PO_4_-SS. As a comparison, the pure Ag_3_PO_4_ with the additives of sodium citrate or sodium stearate are recorded as Ag_3_PO_4_-SC and Ag_3_PO_4_-SS, respectively. Then, the physical and chemical properties of the as-prepared materials were systematically characterized, and their photocatalytic activity and stability under visible light irradiation were also evaluated for DS degradation. Moreover, the effect of H_2_O_2_ on the catalytic property and mechanism of CuBi_2_O_4_/Ag_3_PO_4_ was also comprehensively investigated.

## 2. Materials and Methods

### 2.1. Materials

All reactants and solvents were analytical grade and used without further purification. AgNO_3_, Na_2_HPO_4_·12H_2_O, Bi(NO_3_)_3_·5H_2_O, Cu(NO_3_)_2_·3H_2_O, H_2_O_2_, sodium citrate (Na_3_Cit), sodium stearate (C_17_H_35_COONa), tert-butanol (*t*-BuOH), para-benzoquinone (BZQ), disodium ethylenediaminetetra-acetate (Na_2_-EDTA), ethanol, and diclofenac sodium were obtained from Sinopharm Chemical Reagent Co., Ltd., (Shanghai, China). Ultrapure water was used throughout this study.

### 2.2. Preparation of Hetero-Structural CuBi_2_O_4_/Ag_3_PO_4_

CuBi_2_O_4_ was first synthesized through the hydrothermal synthesis method, and the detailed processes are as follows. Simply speaking, 1.3582 g Bi(NO_3_)_3_·5H_2_O was adequately dissolved into HNO_3_. Next, 20 mL of Cu(NO_3_)_2_·3H_2_O (0.3382 g) was added into the above solution mechanically and agitated for 30 min. Then the precipitator NaOH (1.2 M, 20 mL) was added to the reaction system drop by drop. After the solution was diluted to 70 mL, the mixture was transferred into a sealed Teflon-lined stainless steel autoclave of 100 mL and reacted at 100 °C for 24 h under autogenous pressure. The precipitates were isolated by centrifugation when the autoclave was cooled naturally to room temperature; finally, the solids were washed several times with distilled water and dried at 60 °C for 24 h.

The CuBi_2_O_4_/Ag_3_PO_4_ composites with different hetero-structures were prepared through an in-situ deposition process by adding different surfactants of sodium citrate or sodium stearate into the preparation systems. The detailed preparation process is as following. An amount of 0.1 g of CuBi_2_O_4_ prepared above was first dispersed in 40 mL of ultrapure water and ultrasonically treated at 100 W for 15 min, then 10 mL of sodium citrate (or sodium stearate) solution was added into the reaction system and mechanically agitated for 2 h. After that, 10 mL of AgNO_3_ solution was added into the mixture. After further stirring for 30 min, 20 mL of Na_2_HPO_4_·12H_2_O was added dropwise into the system. The precipitate was isolated and washed several times with absolute ethanol and distilled water, then dried at 60 °C overnight. According to the added surfactants of sodium citrate or sodium stearate, two kinds of CuBi_2_O_4_/Ag_3_PO_4_ can be obtained, and the products can be respectively named as CuBi_2_O_4_/Ag_3_PO_4_-SC (wt:wt = 3:7) and CuBi_2_O_4_/Ag_3_PO_4_-SS (wt:wt = 1:1). As a comparison, the pure Ag_3_PO_4_ with the additives of sodium citrate or sodium stearate are recorded as Ag_3_PO_4_-SC and Ag_3_PO_4_-SS, respectively.

### 2.3. Characterization

Powder X-ray diffraction (XRD, D/MAX-2500/PC) was used to examine the crystalline phase of the products, and each sample was scanned through a 2*θ* range of 10–90° at a rate of 4°/min. The nitrogen adsorption-desorption isotherms of the photocatalysts were obtained using an NOVA-2200e volumetric analyzer (Quantachrome, Boynton Beach, FL, USA), and the surface areas of the samples estimated by the BET model. The morphology of the samples was obtained by scanning electron microscopy (SEM, JSM-6610LV, JOEL, Tokyo, Japan) and high-resolution transmission electron microscopy (HRTEM, Tecnai G^2^ F20 S-TWIN, FEI, Hillsboro, OR, USA). The ultraviolet-visible diffuse reflectance spectra were obtained using a scanning UV-Vis spectrophotometer (UV-2550, Shimadzu, Kyoto, Japan) within a range of 200–800 nm. Additionally, the X-ray photoelectron spectra (XPS, ESCALAB 250, Thermo Scientific, Waltham, MA, USA) were recorded with Al K*α* radiation to investigate the content of Ag^0^ in the fresh and reused catalysts. The photoluminescence (PL) spectroscopy was performed using a florescence spectrophotometer (FP-6500, JASCO, Oklahoma City, OK, USA).

### 2.4. Photocatalytic Performance Measurement

The photocatalytic activity of the as-prepared materials was evaluated for DS degradation, and the reaction apparatus was a photocatalytic reactor (BL-GHX-V, Bilang Biological Science and Technology Co., Ltd., Xi’an, China) using a 300 W Xe lamp with an ultraviolet cutoff filter (providing visible light ≥400 nm) as the light source. In each experiment, 30 mg of photocatalyst was added to a 50 mL DS solution at an initial concentration of 15 mg/L. Prior to illumination, the solution was magnetically stirred in the dark for 30 min to reach the adsorption-desorption equilibrium between the DS and photocatalysts. Then, the solution was exposed to Xe lamp irradiation. At a given time interval of irradiation, one reaction tube was taken out and magnetically separated to remove the catalyst. Finally, the supernatant was withdrawn and filtered with 0.45 µm membrane filters. The concentration of DS and TOC were measured by high efficiency liquid chromatography (HPLC, Agilent 1260, Santa Clara, CA, USA) and a TOC (Shimadzu TOC-V_CPH_, Kyoto, Japan) analyzer, respectively. To determine the effect of H_2_O_2_ on the catalytic performance of CuBi_2_O_4_/Ag_3_PO_4_, different concentrations of H_2_O_2_ were added to the reaction system in the early stage, while the other experimental steps were the same as for the photocatalytic activity. In addition, the repeated experiments for DS degradation were also conducted to study the stability of the as-prepared photocatalysts, and the operation processes were also similar to the photocatalytic experiments.

### 2.5. Analysis of Reactive Species

Free radical capture experiments were used to ascertain the reactive species for DS photodegradation, and tert-butanol (*t*-BuOH) was chose as the hydroxyl radical (OH^•^) scavenger, disodium ethylenediamine tetra-acetate (EDTA-Na_2_) was chose as the hole (*h*^+^) scavenger, benzoquinone (BZQ) was chose as the superoxide radical (O_2_^•−^) scavenger. The detailed free radical capture experiment processes were similar to the photocatalytic activity experiments.

## 3. Results

### 3.1. Characterizations

**Figure 1:**
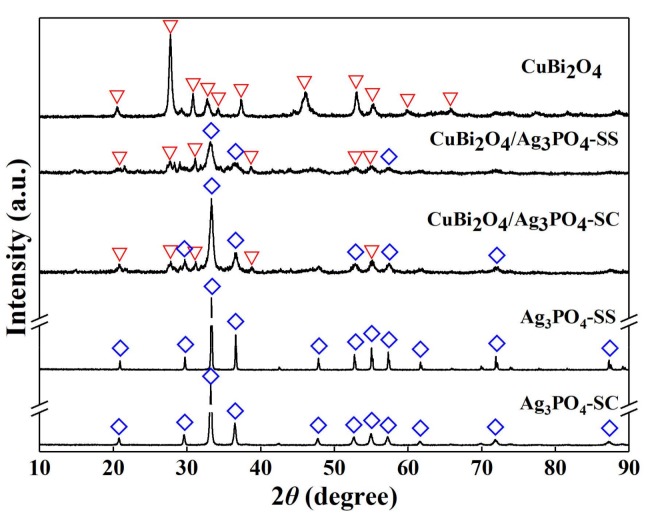
Powder X-ray diffraction (XRD patterns of the as-prepared photocatalysts.

Figure 1 shows the XRD patterns of the synthesized photocatalysts of CuBi_2_O_4_, Ag_3_PO_4_-SC, Ag_3_PO_4_-SS, CuBi_2_O_4_/Ag_3_PO_4_-SC, and CuBi_2_O_4_/Ag_3_PO_4_-SS. It can be seen that the diffraction peaks in the XRD pattern of pure CuBi_2_O_4_ can be perfectly indexed to the phase of CuBi_2_O_4_ (JCPDS No. 84-1969) [26], and the reflections at 2*θ* = 20.48°, 27.73°, 30.76°, 32.73°, 34.20°, 37.30°, 46.04°, 53.02°, 55.08°, 59.94°, and 65.92° are attributed to the crystal planes of (200), (211), (002), (310), (102), (202), (411), (213), (332), (521), and (413), respectively. For the XRD patterns of Ag_3_PO_4_-SC and Ag_3_PO_4_-SS, all the diffraction peaks correspond to the standard card of Ag_3_PO_4_ (JCPDS No.06-0505) [13]. In addition, as can be seen from the XRD patterns of CuBi_2_O_4_/Ag_3_PO_4_-SC and CuBi_2_O_4_/Ag_3_PO_4_-SS, the diffraction peaks only indicate CuBi_2_O_4_ or Ag_3_PO_4_, with absence of any other substances, indicating that Ag_3_PO_4_ couples with CuBi_2_O_4_ mainly through physical effects, but not chemical reaction.

**Figure 2:**
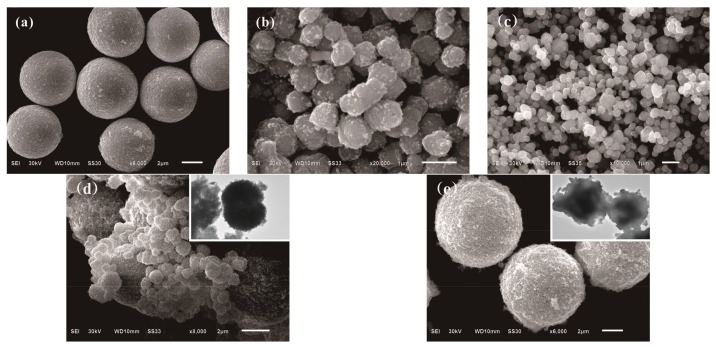
Scanning electron microscopy (SEM) images of photocatalysts, (**a**) CuBi_2_O_4_; (**b**) Ag_3_PO_4_-SC; (**c**) Ag_3_PO_4_-SS; (**d**) CuBi_2_O_4_/Ag_3_PO_4_-SC; (**e**) CuBi_2_O_4_/Ag_3_PO_4_-SS. The illustrations in (**d**,**e**) indicate the high-resolution transmission electron microscopy (HRTEM) images of CuBi_2_O_4_/Ag_3_PO_4_-SC and CuBi_2_O_4_/Ag_3_PO_4_-SS, respectively.

The SEM images of as-prepared photocatalysts of CuBi_2_O_4_, Ag_3_PO_4_-SC, Ag_3_PO_4_-SS, CuBi_2_O_4_/Ag_3_PO_4_-SC, and CuBi_2_O_4_/Ag_3_PO_4_-SS are shown in Figure 2. Apparently, from Figure 2a, pure CuBi_2_O_4_ presented a spherical structure with good dispersibility and smooth surface, its diameter is about 5 µm. Figure 2b displays the micro-topography structure of Ag_3_PO_4_-SC using sodium citrate as additive, and the products exhibit irregular spheres of about 500–800 nm in diameter. If the additive of sodium stearate is used in the preparation system of Ag_3_PO_4_, the product Ag_3_PO_4_-SS shown in Figure 2c behaves with a smaller diameter of about 200–600 nm as irregular spheres. Figure 2d, indicating the SEM and HRTEM images of CuBi_2_O_4_/Ag_3_PO_4_-SC, demonstrates the composite material contains two kinds of spherical structure with different diameters, and the substances are CuBi_2_O_4_ and Ag_3_PO_4_ by comparison with the SEM images of pure CuBi_2_O_4_ and Ag_3_PO_4_-SC. Moreover, a heterojunction has formed due to the close contact between CuBi_2_O_4_ and Ag_3_PO_4_. However, as for the CuBi_2_O_4_/Ag_3_PO_4_-SS, whose SEM and HRTEM images are shown in Figure 2e, only one spherical structure with rough surface and diameter of about 5–6 µm is obtained, indicating the nano particulates Ag_3_PO_4_ are attached onto the surface of CuBi_2_O_4_ and a complete heterojunction composite is formed.

**Figure 3:**
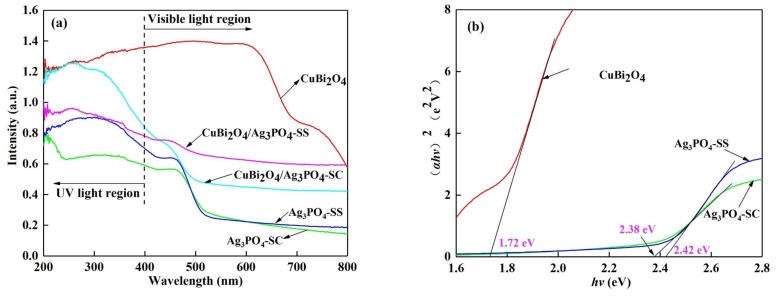
(**a**) UV-Vis diffuse reflectance spectra of the as-prepared materials, (**b**) band gap energy (*E_g_*) of CuBi_2_O_4_, Ag_3_PO_4_-SC, and Ag_3_PO_4_-SS derived from the plots of (*αhv*)^2^ versus energy (*hv*).

The UV-Vis absorption spectra of the as-synthesized samples are shown in Figure 3a. Both pure Ag_3_PO_4_-SC and Ag_3_PO_4_-SS can absorb visible light with wavelengths higher than 500 nm, and pure CuBi_2_O_4_ shows the largest absorbing boundary higher than 800 nm. When the heterojunction structure forms between Ag_3_PO_4_ and CuBi_2_O_4_, the composites CuBi_2_O_4_/Ag_3_PO_4_-SC and CuBi_2_O_4_/Ag_3_PO_4_-SS also exhibit intense absorption bands in the visible-light region. According to the Kubelka-Munk function [27] and the plot of (*αhv*)^2^ vs. *hv* (shown in Figure 3b), the band gaps (Eg) of CuBi_2_O_4_, Ag_3_PO_4_-SC, and Ag_3_PO_4_-SS can be estimated as 1.72 eV, 2.38 eV, and 2.42 eV, respectively. Besides, the band-edge potentials of the conduction band (E_CB_) and valence band (E_VB_) can be designated as [28]:

(1) $$E_{VB}=X-E^{C}+0.5E_{g},$$

and

(2) $$E_{CB}=X-E^{C}-0.5E_{g},$$

where *X* is the geometric mean of the electronegativity of the constituent atoms (5.96 eV for Ag_3_PO_4_, and 4.59 eV for CuBi_2_O_4_ [28,29]), and E^c^ is the energy of the free electrons on the hydrogen scale (approximately 4.5 eV) [28]. Therefore, the E_VB_ and E_CB_ of CuBi_2_O_4_ can be estimated to be 0.95 and −0.77 eV/NHE, the E_VB_ and E_CB_ of Ag_3_PO_4_-SC can be estimated to be 2.65 and 0.27 eV/NHE, while the E_VB_ and E_CB_ of Ag_3_PO_4_-SS are estimated to be 2.67 and 0.25 eV/NHE, respectively.

### 3.2. Photocatalytic Activity for DS Degradation

**Figure 4:**
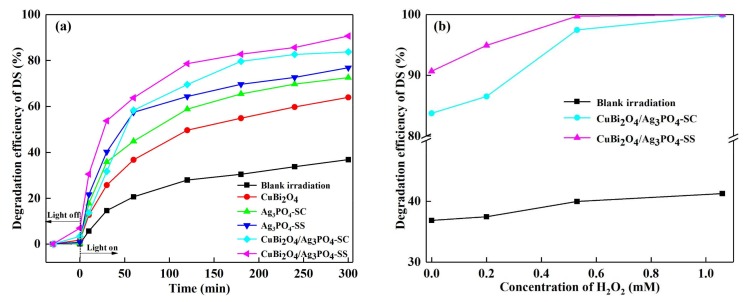

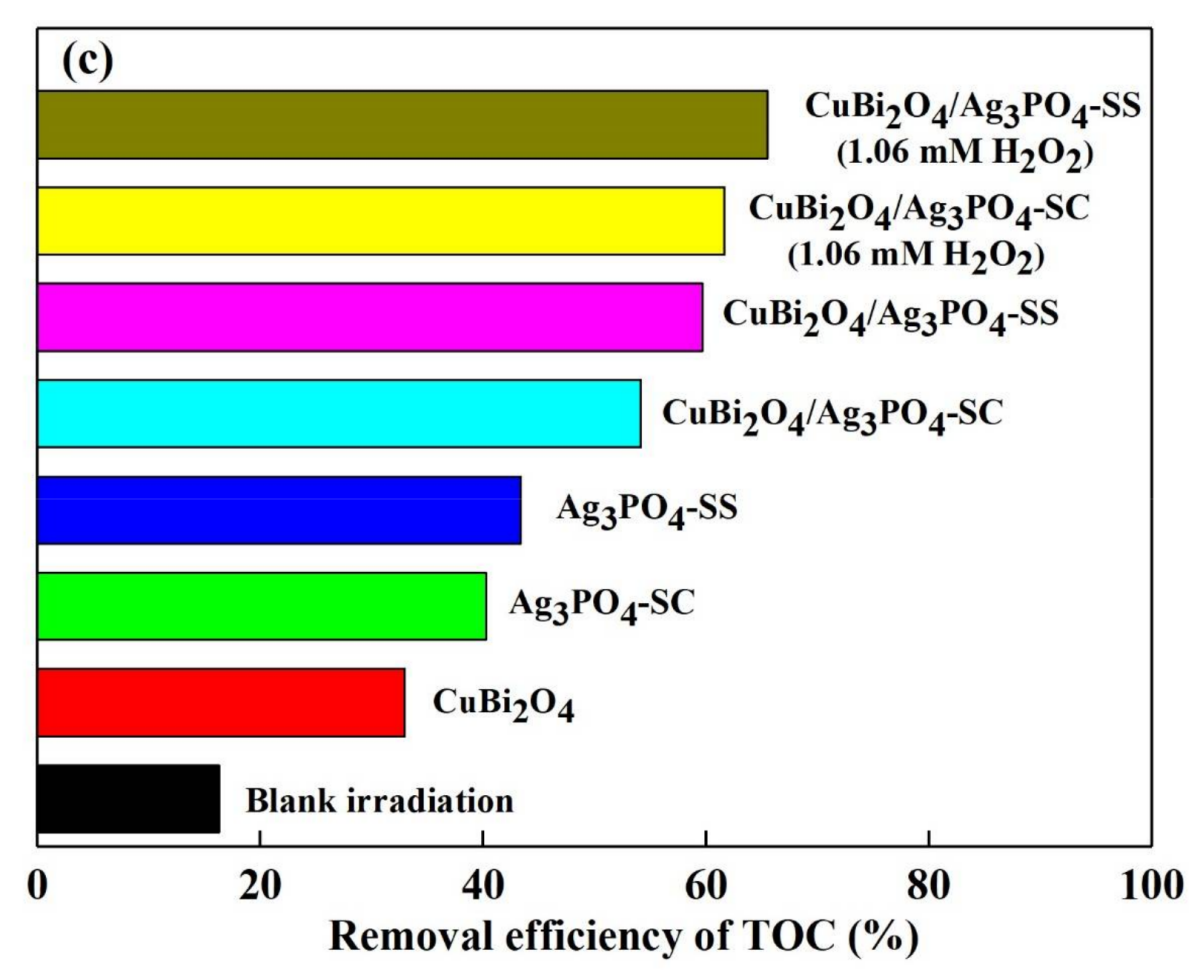
(**a**) Degradation efficiency of diclofenac sodium (DS) over different photocatalysts; (**b**) effect of H_2_O_2_ concentration on the removal efficiency of DS over CuBi_2_O_4_/Ag_3_PO_4_-SC and CuBi_2_O_4_/Ag_3_PO_4_-SS; (**c**) removal efficiency of TOC under different photocatalyst systems.

To evaluate the photocatalytic activity of the as-prepared catalysts, DS was selected as the target pollutant. Figure 4a shows the degradation efficiency of DS in different photocatalyst systems. In the system without any other catalysts, the degradation efficiency of DS is 36.87% under 300 min of visible light irradiation. As for the prepared catalysts, their adsorption efficiencies in the dark for DS are 1.76% (CuBi_2_O_4_), 0.30% (Ag_3_PO_4_-SC), 0.77% (Ag_3_PO_4_-SS), 3.36% (CuBi_2_O_4_/Ag_3_PO_4_-SC), and 6.98% (CuBi_2_O_4_/Ag_3_PO_4_-SS), respectively. This adsorption rule of catalysts is consistent with their specific surface area characteristics, whose data are displayed in Table 1; the specific surface area is an important parameter to evaluate the active adsorption sites of materials. Thus, the relatively lower adsorption efficiencies of as-prepared catalysts toward DS suggests the final removal rate of DS in the catalysts’ system is mainly due to the photocatalysis. Under the same conditions, the degradation efficiencies of DS in CuBi_2_O_4_, Ag_3_PO_4_-SC, Ag_3_PO_4_-SS, CuBi_2_O_4_/Ag_3_PO_4_-SC, and CuBi_2_O_4_/Ag_3_PO_4_-SS systems are 63.98%, 72.55%, 76.87%, 83.75% and 90.68%, respectively. These results indicate that the addition of catalysts improves the photodegradation of DS and there is a synergy between CuBi_2_O_4_ and Ag_3_PO_4_.

Figure 4b shows the degradation efficiency of DS in different photocatalytic systems in the presence of H_2_O_2_. Overall, the degradation efficiency of DS increases with the increase of H_2_O_2_ concentration. When the concentration of H_2_O_2_ is 1.06 mM, the removal rate of DS in the blank irradiation system is 41.26%, which only improves by 4.39% of that without H_2_O_2_ (36.87%), showing no obvious effect of pure H_2_O_2_ on DS photodegradation. While for the CuBi_2_O_4_/Ag_3_PO_4_-SC and CuBi_2_O_4_/Ag_3_PO_4_-SS photocatalysts systems, the removal rates of DS achieve 97.48% and 99.73% when the concentration of H_2_O_2_ is 0.53 mM; in addition, the DS can be completely degraded when the concentration of H_2_O_2_ further increases to 1.06 mM. This reveals that there are synergistic effects between H_2_O_2_ and CuBi_2_O_4_/Ag_3_PO_4_ on the DS photodegradation.

In order to further demonstrate the synergistic promotion between CuBi_2_O_4_ and Ag_3_PO_4_, as well as H_2_O_2_ and CuBi_2_O_4_/Ag_3_PO_4_ for DS photodegradation, the mineralization rates of DS in the single CuBi_2_O_4_ or Ag_3_PO_4_ system, CuBi_2_O_4_/Ag_3_PO_4_ composite system, and the simultaneous presence system of H_2_O_2_ and CuBi_2_O_4_/Ag_3_PO_4_ were investigated. The experimental results are shown in Figure 4c. It can be clearly seen that mineralization rates of 16.27%, 32.97%, 40.23%, and 43.36% are obtained in the blank illumination, single CuBi_2_O_4_, Ag_3_PO_4_-SC, and Ag_3_PO_4_-SS systems, respectively. While under the same conditions, the mineralization rates of DS in CuBi_2_O_4_/Ag_3_PO_4_-SC and CuBi_2_O_4_/Ag_3_PO_4_-SS systems are 54.15% and 59.68%, respectively, which is an increase of 13.9% compared with that of the single catalyst system. In addition, the mineralization rates of DS in the CuBi_2_O_4_/Ag_3_PO_4_-SC and CuBi_2_O_4_/Ag_3_PO_4_-SS systems are further increased by 7.48% and 5.81% when the H_2_O_2_ concentration is 1.06 mM. These experimental results show the improvement of catalytic activity of CuBi_2_O_4_/Ag_3_PO_4_ composite in contrast with the single catalyst, and the further promotion effect of H_2_O_2_ on the catalytic activity of CuBi_2_O_4_/Ag_3_PO_4_ composite.

### 3.3. Photocatalytic Stability of the Catalysts

**Figure 5:**
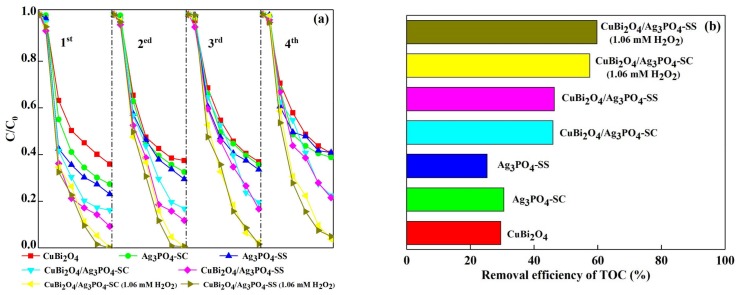
(**a**) Degradation efficiency of DS with different photocatalysts in different recycle runs; (**b**) TOC removal efficiency of the DS solution with four times recycling of photocatalysts.

The stability of a photocatalyst is an important index to evaluate the value of its research value and actual utilization [30]. Therefore, we investigated the photocatalytic stability of CuBi_2_O_4_, Ag_3_PO_4_-SC, Ag_3_PO_4_-SS, CuBi_2_O_4_/Ag_3_PO_4_-SC, CuBi_2_O_4_/Ag_3_PO_4_-SS, CuBi_2_O_4_/Ag_3_PO_4_-SC (H_2_O_2_, 1.06 mM) and CuBi_2_O_4_/Ag_3_PO_4_-SS (H_2_O_2_, 1.06 mM) using repeated experiments. The results described in Figure 5a show that the removal rates of DS in CuBi_2_O_4_, Ag_3_PO_4_-SC, and Ag_3_PO_4_-SS systems decrease by 4.62%, 11.42%, and 17.80% after four times recycling, indicating the poor stability of the single photocatalysts. While in the CuBi_2_O_4_/Ag_3_PO_4_-SC and CuBi_2_O_4_/Ag_3_PO_4_-SS composite systems, the removal rates of DS decrease by 6.31% and 12.32%, respectively. Compared with the single Ag_3_PO_4_, the stability of the composite photocatalysts are improved to some extent. In addition, though the removal rates of DS are still decreased (decreased by 3.65% and 3.43%) when 1.06 mM H_2_O_2_ was added to the CuBi_2_O_4_/Ag_3_PO_4_-SC and CuBi_2_O_4_/Ag_3_PO_4_-SS systems, 96.24% and 95.02% of removal rates were obtained, which indicates that the addition of H_2_O_2_ greatly enhances the stability of CuBi_2_O_4_/Ag_3_PO_4_. The results depicted in Figure 5b show that the mineralization rates of DS for the fourth time recycled CuBi_2_O_4_, Ag_3_PO_4_-SC, Ag_3_PO_4_-SS, CuBi_2_O_4_/Ag_3_PO_4_-SC, CuBi_2_O_4_/Ag_3_PO_4_-SS, CuBi_2_O_4_/Ag_3_PO_4_-SC (H_2_O_2_, 1.06 mM), and CuBi_2_O_4_/Ag_3_PO_4_-SS (H_2_O_2_, 1.06 mM) systems are 29.49%, 30.51%, 25.27%, 45.93%, 46.36%, 57.47%, and 59.73%, respectively. Compared with pure Ag_3_PO_4_, it is a fact that the stability of the composite photocatalyst CuBi_2_O_4_/Ag_3_PO_4_ is improved, and the addition of H_2_O_2_ is helpful to further improve the photocatalytic stability of CuBi_2_O_4_/Ag_3_PO_4_.

**Figure 6:**
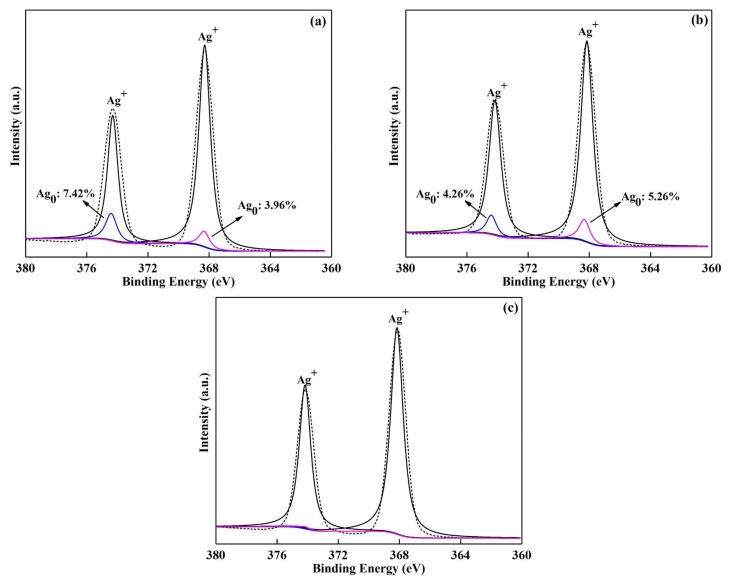
X-ray photoelectron spectra of Ag3d of the recycled photocatalysts, (**a**) Ag_3_PO_4_-SC; (**b**) CuBi_2_O_4_/Ag_3_PO_4_-SC; (**c**) CuBi_2_O_4_/Ag_3_PO_4_-SC (1.06 mM H_2_O_2_).

The instability of the catalysts is mainly caused by the reduction of Ag^+^ from the lattice of Ag_3_PO_4_ to generate Ag^0^ during the reaction process. In order to analyze the photocatalytic stability of the as-prepared catalysts from the perspective of structure itself, XPS analysis of Ag3d for the fresh and reused catalysts was performed and the results are shown in Figure 6. It can be seen that, except for the Ag^+^ peaks (374.17 eV and 368.16 eV) in the reused catalysts of Ag_3_PO_4_-SC and CuBi_2_O_4_/Ag_3_PO_4_-SC, peaks indicating Ag^0^ (374.40 eV and 368.32 eV) [31] are also observed, and the proportion of the peaks relative to Ag^0^ occupy 11.38% and 9.52% of the total fitting peak area in the two catalysts. However, no obvious peaks relative to Ag^0^ exist in the catalyst of CuBi_2_O_4_/Ag_3_PO_4_-SC when 1.06 mM H_2_O_2_ was added into the reaction system. These results imply the enhanced stability of CuBi_2_O_4_/Ag_3_PO_4_ in comparison to pure Ag_3_PO_4_, and again the further promotion of H_2_O_2_ for the composite’s stability.

### 3.4. Photocatalytic Mechanism Discussion

**Figure 7:**
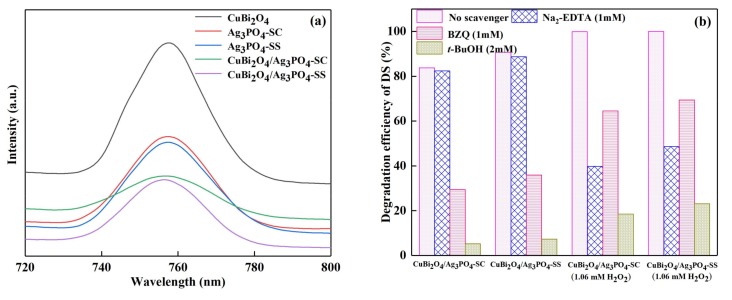
(**a**) Photoluminescence (PL) emission spectra of the as-prepared catalysts; (**b**) degradation efficiency of DS over different catalyst systems in the presence of scavengers.

Photoluminescence (PL) emission spectroscopy is important for understanding the photocatalytic mechanism of a catalyst by analyzing the migration and separation efficiency of photogenerated charge carriers in the material. Also, lower PL emission intensity implies a lower electron-hole recombination rate and corresponds to higher photocatalytic activity. Thus in this study, the PL emission spectra of the as-prepared catalysts were recorded under excitation at 500 nm, and the results are shown in Figure 7a. Compared with the pure CuBi_2_O_4_, Ag_3_PO_4_-SC, and Ag_3_PO_4_-SS, the composites CuBi_2_O_4_/Ag_3_PO_4_-SC and CuBi_2_O_4_/Ag_3_PO_4_-SS exhibit decreased emission intensity, suggesting the formation of the heterojunction between CuBi_2_O_4_ and Ag_3_PO_4_ decreases the recombination rate of electron-hole pairs. Moreover, the PL emission intensity rule of these catalysts is seriously coincident with that of the photocatalytic activity of the as-prepared materials.

Active species are important participants in photochemical reactions and they are also the key to discuss the photocatalytic mechanism. Thus, the free radical capture experiments were used to investigate whether OH^•^, *h*^+^, and O_2_^•−^ are involved in the DS photodegradation process as well as their contribution order, while tert-butanol (*t*-BuOH), disodium ethylenediaminetetra-acetate (EDTA-Na_2_), benzoquinone (BZQ) were chosen as the hydroxyl radical (OH^•^), hole (*h*^+^), superoxide radical (O_2_^•−^) scavengers, respectively. The experiment results are shown in Figure 7b. For the CuBi_2_O_4_/Ag_3_PO_4_-SC and CuBi_2_O_4_/Ag_3_PO_4_-SS photocatalyst systems, the degradation efficiency of DS changed little when 1 mM Na_2_-EDTA was added; while the degradation efficiency of DS decreased by 54.29% and 54.76% in the two catalyst systems when 1mM BZQ was added; when 2 mM *t*-BuOH was added, the degradation efficiency of DS decreased by 78.58% and 83.34%. This indicates that *h*^+^ does not participate in the degradation of DS in the CuBi_2_O_4_/Ag_3_PO_4_ photocatalyst systems, the active species involved in DS degradation are OH^•^ and O_2_^•−^, and the contribution order is OH^•^ >O_2_^•−^. Based on a similar analysis mechanism, in the CuBi_2_O_4_/Ag_3_PO_4_-SC and CuBi_2_O_4_/Ag_3_PO_4_-SS photocatalysts systems with H_2_O_2_, *h*^+^, OH^•^, and O_2_^•−^ all involved in the degradation of DS, the contributions order is OH^•^ > *h*^+^> O_2_^•−^.

**Figure 8:**
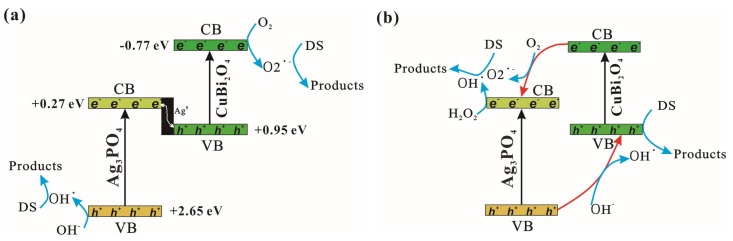
Schematic diagram for *E*g matching and flow of photoinduced electrons/holes for (**a**) CuBi_2_O_4_/Ag_3_PO_4_-SC; (**b**) CuBi_2_O_4_/Ag_3_PO_4_-SC (H_2_O_2_).

Based on the above analysis, the photocatalytic mechanism of CuBi_2_O_4_/Ag_3_PO_4_ with or without H_2_O_2_ was discussed in detail. The following investigation only focuses on the catalyst CuBi_2_O_4_/Ag_3_PO_4_-SC prepared using sodium citrate as the additive to discuss the effect of H_2_O_2_ on the photocatalytic mechanism of CuBi_2_O_4_/Ag_3_PO_4_. Figure 8a shows the migration pathways of the photogenerated electrons and holes of CuBi_2_O_4_/Ag_3_PO_4_ without H_2_O_2_ under visible light irradiation. As can be seen from the XPS spectrum of recycled CuBi_2_O_4_/Ag_3_PO_4_ (Figure 6b), Ag^0^ has been formed on the surface of CuBi_2_O_4_/Ag_3_PO_4_ after reaction. Thus, the formed Ag^0^ becomes the recombination center of *h*^+^ coming from the valence band of CuBi_2_O_4_ and the *e*^−^ from the conduction band of Ag_3_PO_4_, thereby promoting the separation of photogenerated *h*^+^-*e*^−^ pairs. In addition, *e*^−^ accumulated at the conduction band of CuBi_2_O_4_ can induce the generation of O_2_^•−^, and *h*^+^ accumulated at the valence band of Ag_3_PO_4_ can induce the generation of OH^•^ (*E*(OH^−^/OH^•^) = 1.99 eV [32]. The results of the free radical capture experiment show that the contribution of *h*^+^ to DS degradation is small in the photocatalytic system using CuBi_2_O_4_/Ag_3_PO_4_ as catalyst, indicating that *h*^+^ is mainly involved in the production of OH^•^ and not involved in the degradation of DS. Therefore, the photocatalytic mechanism of CuBi_2_O_4_/Ag_3_PO_4_ is consistent with the Z-scheme theory described by Yu et al. [17,33] But as can be seen from Figure 8b, the photocatalytic mechanism of CuBi_2_O_4_/Ag_3_PO_4_ in the presence of H_2_O_2_ is more suitable to be explained by the heterojunction band theory [34]. That is, the electrons in the CB of CuBi_2_O_4_ can be transferred to the CB of Ag_3_PO_4_ easily, while the holes in the VB of Ag_3_PO_4_ can be transferred to the VB of CuBi_2_O_4_ due to the fact that both of the CB and VB positions of CuBi_2_O_4_ are higher than that of the Ag_3_PO_4_, which promotes the separation of photogenerated *h*^+^-*e*^−^ pairs. Meanwhile, in the migration process of the photogenerated holes, the OH^−^ can be selectively oxidized to OH^•^ by the holes (*E*_VB_ = 2.65 eV), which is due to the higher energy of holes than the standard redox potential of *E*(OH^−^/OH^•^) = 1.99 eV (vs. NHE); while in the migration process of the photogenerated electrons, the O_2_ adsorbed on the catalyst surface can be induced to generate O_2_^•−^ [35], meanwhile, the *e*^-^ transforming into CB of Ag_3_PO_4_ (*E*_CB_ = 0.27 eV) can be easily be captured by the additive H_2_O_2_ for the generation of OH^•^ (*E*(H_2_O_2_/OH^•^) = 0.38 eV) [36]. Therefore, OH^•^ becomes the most important participant in DS degradation owing to its multi-channel sources, which is consistent with the results of the free radical capture experiment. In summary, the enhanced photocatalytic activity of CuBi_2_O_4_/Ag_3_PO_4_ by H_2_O_2_ is mainly due to the splitting of H_2_O_2_ to form much more effective OH^•^ to degrade DS; and the enhanced stability of CuBi_2_O_4_/Ag_3_PO_4_ in the present of H_2_O_2_ is owing to the effective capture of photogenerated *e*^−^ by H_2_O_2_, which greatly inhibits the reduction of Ag^+^ from the lattice of Ag_3_PO_4_.

## 4. Conclusions

In this paper, two kinds of hetero-structural composite photocatalysts CuBi_2_O_4_/Ag_3_PO_4_ were prepared using sodium citrate or sodium stearate as additives. Under the same conditions, both of the two kinds of CuBi_2_O_4_/Ag_3_PO_4_ composites showed much improved excellent photocatalytic activity and stability than the single Ag_3_PO_4_. Moreover, the addition of H_2_O_2_ further enhanced the photocatalytic performance of CuBi_2_O_4_/Ag_3_PO_4_. From the free radical capture experiments, the active species involved in DS degradation in the pure CuBi_2_O_4_/Ag_3_PO_4_ system are OH^•^ and O_2_^•−^, and the contribution order is OH^•^ >O_2_^•−^; while as H_2_O_2_ is added into the CuBi_2_O_4_/Ag_3_PO_4_ photocatalytic system,* h*^+^, OH^•^, and O_2_^•−^ are all involved in the degradation of DS, and the contribution order is OH^•^ > *h*^+^ > O_2_^•−^. Therefore, the catalytic mechanism of CuBi_2_O_4_/Ag_3_PO_4_ conforms to the Z-scheme theory; while the mechanism changes to heterojunction band theory after the addition of H_2_O_2_.

## Figures and Tables

**Table 1:** Specific surface area of the as-prepared catalysts.

Samples	BET Surface Area (m^2^ g^−1^)
CuBi_2_O_4_	2.307
Ag_3_PO_4_-SC	0.406
Ag_3_PO_4_-SS	0.984
CuBi_2_O_4_/Ag_3_PO_4_-SC	12.218
CuBi_2_O_4_/Ag_3_PO_4_-SS	14.596
